# The Association of Sleep Quality and Vitamin D Levels in Hemodialysis Patients

**DOI:** 10.1155/2021/4612091

**Published:** 2021-09-21

**Authors:** Seyyedeh Mina Hejazian, Elham Ahmadian, Sepideh Zununi Vahed, Leili Faraji Gogani, Farahnoosh Farnood

**Affiliations:** ^1^Kidney Research Center, Tabriz University of Medical Sciences, Tabriz, Iran; ^2^Student Research Committee, Tabriz University of Medical Sciences, Tabriz, Iran; ^3^Faculty of Medicine, Islamic Azad University of Tabriz Branch, Tabriz, Iran; ^4^Liver and Gastrointestinal Diseases Research Center, Tabriz University of Medical Sciences, Tabriz, Iran

## Abstract

**Background:**

To date, hemodialysis (HD) is the most common therapy for chronic kidney disease (CKD) patients. However, it causes different complications such as sleep disorders. Sleep regulation is connected to vitamin D; hence, its deficiency might influence the quality and duration of sleep. This study is aimed at evaluating the correlation of sleep quality and vitamin D levels in 80 HD patients.

**Methods:**

This cross-sectional study was performed on 80 hemodialysis patients admitted to 29 Bahman hospitals in Tabriz, Iran. Before beginning of dialysis, serum 25 (OH) D levels were assessed among patients and the sleep patterns and sleep quality of patients were accurately calculated by the Pittsburgh sleep quality index (PSQI) standard questionnaire.

**Results:**

Our results showed that 22 HD patients (27.5%) had severe sleep disorders. In addition, it was found that serum levels of vitamin D had significant correlation with sleep quality (*r* = −0.341, *p* = 0.002) in general, even after adjusting confounding factors such as calcium (Ca), phosphate (P), and parathyroid hormone (PTH) level. In poor sleepers (PSQI ≤ 5), a negative correlation was observed between the levels of vitamin D and PSQI score (*r* = −0.397, *p* = 0.004). PSQI scores in the normal range of PTH (*r* = −0.377, *p* = 0.006) and in >600 pg/ml of PTH (*r* = −0.675, *p* = 0.011) had a correlation with vitamin D levels. The level of vitamin D was the single independent predictor of sleep efficiency (*β* coefficient = −0.386, *p* = 0.001).

**Conclusion:**

The present project reported that the positive effect of vitamin D is associated with sleep disorder in HD patients. In future studies, normal levels of Ca and P should be considered along with normal vitamin D levels among the included patients.

## 1. Introduction

Hemodialysis (HD) is the main therapy for patients with chronic kidney disease (CKD) that causes health problems [1]. Sleep disorders are common complications in HD patients affecting about 41-85% of them [2, 3]. As an emergent risk factor, sleep disturbance impacts different aspects of HD patients and it can predict their life quality and mortality [2]. Moreover, by altering innate and cellular immunity, sleep disorder is connected with inflammation and inflammatory disorders including infection, diabetes, cardiovascular disease, and autoimmune disease [4, 5]. Different factors such as hypertension, morning dialysis shift, blood gases, blood creatinine and urea, anemia, and stressful life are putative causes for sleep disturbance [6–8]. Moreover, higher levels of calcium (Ca), phosphate (P), and parathyroid hormone (PTH) and decreased levels of vitamin D impair sleep quality in patients undergoing hemodialysis [9].

Beyond its function on calcium-phosphate metabolism, vitamin D exerts several pleiotropic effects [10]. Vitamin D regulates sleep directly and indirectly, and mounting evidence recommends a connotation between sleep disorders and hypovitaminosis D [11]. Regarding this association, deficient vitamin D (<20 ng/dl 25 (OH) D) is strictly connected with sleep difficulties [12]. Moreover, short sleep duration is associated with 25 (OH) D levels [13, 14].

The exact underlying mechanism of vitamin D deficiency-related sleep problems is unclear. According to the available studies, the expression of vitamin D receptors and their related degradation and activation enzymes (e.g., CYP27B1) happens in the specific regions of the brain that is responsible for sleep regulation. Hence, vitamin D seems to participate in the control of the sleep-wake cycle [15, 16]. Moreover, vitamin D plays a key role in the modulation of the immune system and liberation of proinflammatory cytokines including tumor necrosis factor-*α* (TNF-*α*), IL-1*β*, and IL-6 [17, 18] that are elevated in HD patients [19, 20]. The aforementioned cytokines are involved in sleep regulation [13, 21]; therefore, they may impact the sleep quality of these patients. Inadequate vitamin D can also lead to an elevation in circulating prostaglandin D2 (PD2), another controller of sleep [22, 23]. Furthermore, vitamin D controls the conversion of tryptophan into 5-hydroxytryptophan that is metabolized to serotonin to yield melatonin [24]. The production of sleep hormone melatonin from the pineal gland regulates sleep and circadian rhythms [25, 26].

Since vitamin D deficiency [27] and sleep disorders [28] are two important risk factors of mortality among HD patients, vitamin D deficiency can be restored by prescribing oral vitamin D supplements [29–31]; it is important to manage vitamin D levels to improve the sleep quality of HD patients. In so doing, this study is aimed at determining the effect of vitamin D on the sleep quality of patients under HD.

## 2. Materials and Methods

**Figure 1:**
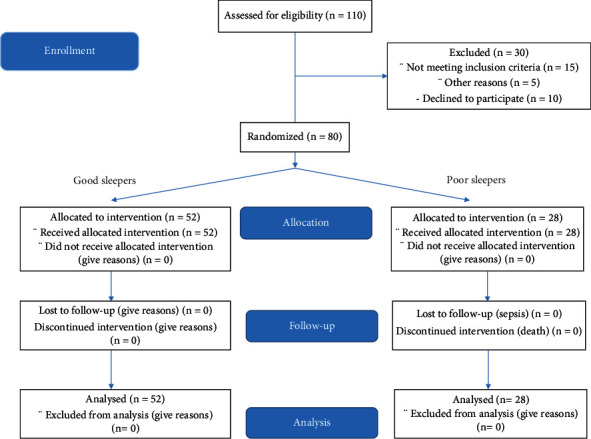
The CONSORT 2010 flow diagram for HD patients.

This cross-sectional study was performed since September 2019 to November 2019 for 4 months among 29 Bahman hospital referees on 110 HD patients. Inclusion criteria were age group of 18-75 years old, at least 6 months after the beginning of dialysis, or <9.5 g/dl hemoglobin (Hb) and tendency to participate in this study. Patients with major depression, severe pulmonary diseases, and acute heart failure were excluded from the study. After considering including and excluding criteria, 80 patients were included in the study (other information is given in detail in Figure 1).

Serum levels of 25 (OH) D were evaluated in patients before dialysis by the immunoassay kits as standard unit of ng/ml, and patients were divided into 4 groups based on Endocrine Society guidelines: 20 ng/ml: deficiency, 21-29 ng/ml: insufficiency, 30-100: sufficiency, and >100 ng/ml: possible harm [32–34]. Based on the vitamin D standardization program (VDSP) in 2010, it is aimed at standardizing vitamin D measurement by reference [35].

Besides, the Pittsburgh sleep quality index (PSQI) standard questionnaire was obtained from http://www.opapc.com/uploads/documents/PSQI.pdf and translated to Persian; its validation was previously confirmed by Zahed et al. [36], and sensitivity and specificity of this questionnaire were determined by Buysse et al. [37]. The sleep patterns and quality of patients were calculated accurately through the PSQI questionnaire that was orally collected from the patients. The questionnaire form was validated and contains 7 items including sleep quality, sleep delay, efficiency, duration, stage disorders, use of sleeping pills, and daily dysfunction. Sleep quality over the past month including duration of sleep, normal bedtime habits, sleep problems such as snoring at night and restless leg syndrome, and the need to take medication before bed was also assessed. A global PSQI score was yielded between 0 and 21 results from the sum of each component (scored from 0 to 3). A PSQI score of zero means no problem, and a score of 21 means a severe problem arising from sleep quality.

### 2.1. Statistical Analysis

Data were analyzed via SPSS version 23. The normality of clinical parameters was assessed by the Shapiro-Wilk test. The clinical and demographic characteristics of patients were shown as the mean ± SD (standard deviation) or median (IQR) (interquartile range). Variables with normal distribution were analyzed by *t*-test, while nonnormal variables were analyzed with the Mann–Whitney *U* test. The evaluation of correlations between the variables was performed by the chi-square or Pearson test. Multivariate linear regression analysis models were performed to assay the impact of the independent variables including age, weight, Hb, PTH, Ca, P, calcium multiplied by phosphate (Ca × P), and vitamin D on sleep efficiency. Odds ratios (OR) were obtained using linear regression with 95% CIs (confidence intervals).

## 3. Results

In this study, serum levels of 25 (OH) D were assessed in 80 HD patients and its association with PSQI score, obtained from a unit global questionnaire, was evaluated. PSQI score was considered as an indicator for determination of sleep quality. The mean age of participants was 59.19 ± 13.51 years old; 27 patients (33.8%) were women and 53 patients (66.3%) were men. The serum levels of 25 (OH) D (ng/ml), Ca, and P were 23.40 ± 12.54, 8.79 ± 0.70, and 4.75 ± 1.04, respectively. Types of vascular access used for HD procedures were nonhomogeneous among patients; 66.3% of which had an arteriovenous fistula (AV fistula) (*n* = 53), 32.5% had central venous catheter (CVC) (*n* = 26), and one patient had arteriovenous graft (AV graft) (1.3%). Furthermore, the incidence of underlying diseases for end-stage renal disease (ESRD) was assessed among patients. It was defined that two patients (1.3%) had autosomal dominant polycystic kidney disease (ADPKD), and three patients (2.5%) had chronic interstitial nephritis. In addition, 57.5% of the patients (*n* = 46) suffered from hypertension (HTN) and 27 patients (33.8%) had HTN and diabetes.

Our results declared that 22 out of 80 HD patients had severe sleep disorders and its incidence was 27.5%. Besides, there was a negative significant correlation between serum levels of vitamin D and sleep quality (*r* = −0.341, *p* = 0.002). There was a statistically significant relationship between serum levels of 25 (OH) D, sleep quality, and control of other confounding variables. It means that the higher serum 25 (OH) D levels, the higher quality of sleep (decreased PSQI score) in these patients. However, observations showed no correlation between age and sleep disorders of patients (*p* = 0.20) and there was no correlation between sleep quality and gender (*p* = 0.396).

HD patients were categorized based on the PSQI score with a cutoff value of 5 [38, 39]. Patients with a PSQI score of equal or less than 5 were considered as good sleepers (21 men, 31 women), while cases with a global PSQI score of more than 5 were considered as poor sleepers (8 men, 20 women). The poor sleeper group in more than 3 areas had moderate problems or in at least 2 areas had severe complications [37]. As presented in Table 1, no meaningful differences for age; gender; Ca, P, Hb, and PTH levels; mode of HD; and causes of ESRD were observed between poor and good sleepers (*p* > 0.05). Moreover, no statistically significant associations were seen between PSQI scores and the studied parameters.

Since serum levels of PTH, Ca, and P can be considered as intervening factors, in the next steps, we classified the patients based on their PTH and Ca × P levels. The normal range of PTH was considered to be between 150 and 600 pg/ml. Based on the PTH level, patients were divided into 3 groups including patients with PTH < 150 pg/ml (*n* = 9), patients with PTH of 150-600 pg/ml (*n* = 51), and patients with PTH > 600 pg/ml (*n* = 13). There was a negative correlation between serum 25 (OH) D and PSQI scores that was statistically significant in the normal range of PTH (*r* = −0.377, *p* = 0.006) and in >600 pg/ml (*r* = −0.675, *p* = 0.011) of PTH. Moreover, a negative correlation was seen between vitamin D and PSQI score in PTH level less than 150 pg/ml; however, it was not statistically significant (*r* = 0.461, *p* = 0.211).

Based on the 45 mg^2^/dl^2^ cutoff point of Ca × P multiplication (Ca × P), participants were also divided into 2 groups. It was revealed that in patients with Ca × P less than 45, there was a significant negative correlation between vitamin D levels and PSQI (*r* = −0.341, *p* = 0.002), but this negative correlation was not statistically significant in patients with Ca × P ≥ 45 (*r* = −0.245, *p* = 0.173).

In multiple linear regression analysis, vitamin D level was the single independent predictor of sleep efficiency (*β* coefficient = −0.386, *p* = 0.001). Moreover, level of vitamin D with OR = 0.970 (*B* = −0.031, CI (0.935-1.00)) was a protective factor for poor sleepers; it was not statistically significant though (*p* = 0.106). Moreover, there was a meaningful correlation between levels of vitamin D and PSQI score in poor sleepers (*r* = −0.397, *p* = 0.004) and between some other variables (Table 2).

In order to assess the effect of vitamin D level on PSQI score, linear regression was used to control the PTH and C × P levels. Based on the regression results, the effects of PTH and C × P levels on PSQI score were not significant; however, the vitamin D levels on PSQI score were statistically significant (*p* = 0.001). Therefore, one by one increasing in PSQI score is along with decreasing 0.16 in average amount of vitamin D (Table 3).

## 4. Discussion

Our study demonstrated a significant association between sleep disorders and serum levels of vitamin D in HD patients, even after adjusting confounding factors such as Ca, P, and PTH level, which is parallel to the results of prior researches in patients with SLE and elderly adults [40–42].

HD patients usually show vitamin D deficiency (as previously mentioned, the cutoff point for vitamin D deficiency is <20 ng/dl) as an independent marker of disease progression and mortality [43, 44], where calcipherol therapy might enhance serum 25 (OH) D levels in HD patients. Exogenous supplementation of vitamin D has been safe and effective in patients undergoing HD [45, 46]. The findings of our study showed that by increasing the serum level of vitamin D, the score obtained from the PSQI questionnaire in these patients decreases, and as a result, the quality of sleep was improved. Our result was in agreement with the result of Çakır et al. [47]. Therefore, it could be said that vitamin D deficiency might be directly linked to sleep disorders in HD patients.

Han et al. showed that most HD cases (62.4%) had sleep disturbance to vitamin D deficiency [48]. However, the accurate prevalence of sleep disorders in HD patients is associated with several factors such as different study designs in the evaluation of sleep disturbances, race/ethnicity, and patient recruitment sources [13]. It has been reported that higher P and PTH and lower Hb and albumin levels are potential risk factors of sleep problems in HD patients [49, 50]. In severe kidney hyperparathyroidism, PTH may play a causative role for sleep disorders and parathyroidectomy could improve the sleep quality in half of the HD cases [51]. In the present study, a statistically significant negative association was detected between serum levels of 25 (OH) D and PSQI scores in patients with ≥150 pg/ml of PTH.

It has been shown that 25 (OH) D deficiency is an important risk factor for reducing sleep quality and the result is independent of other factors such as age, gender, depression, and the effect of hemodialysis [52]. Jean and coworkers showed that a daily dose of 10-30 mg of 25 (OH) D oral supplements completely resolves vitamin D deficiency in HD patients with high safety. The major impacts perceived in this study included improvement of excessive bone turnover, in spite of lower alfacalcidol administration, and elevation in serum albumin level and also in the percentage of patients with serum calcium and phosphorus levels within the recommended protocols [53]. The results of a large study on HD patients in the United States showed that decreased serum levels of 1,25 (OH)_2_D and 25 (OH) D were related to mortality in the first 90 days of hemodialysis. It was independent of regular nutritional factors, kidney function, simultaneous diseases, other predictors of dialysis-related mortality, and most prominently the mineral metabolism biomarkers [54]. In the present study, vitamin D level was shown to be a potential single independent predictor of sleep efficiency that was in agreement with other studies [38, 39].

Therefore, our results showed that deficiency of serum levels of vitamin D could lead to sleep deregulation independent of PTH alterations in HD patients. Nevertheless, a negative correlation between serum levels of PTH and sleep score was not statistically significant in patients with Ca × P more than 45 mg^2^/dl^2^. So, it can be concluded that for modification of quality and quantity of sleep in HD patients, beyond preserving enough vitamin D levels, it should be simultaneously preceded for balancing of Ca and P levels.

Some of the limitations of the current study could be noted. This study should be performed in populations of other races and ethnicities to confirm the final results since the majority of our participants belonged to Azerbaijanis ethnicity in Iran. It is also proposed to consider the diet used by HD patients and their lifestyle during the study. Moreover, objective sleep tests such as multiple sleep tests or polysomnography will be very helpful in proving the results of the questionnaire.

## 5. Conclusion

The present project reported the positive effect of vitamin D on sleep disorders in HD patients, which was based on the results of a questionnaire. This study states that all HD patients with sleep disorders should be carefully assessed for vitamin D status. Moreover, for modification of quality and quantity of sleep in HD patients, in addition to the presence of enough vitamin D levels, it should be simultaneously proceeded for balancing of Ca and P levels.

## Figures and Tables

**Table 1:** Baseline and clinical characteristics between good and poor sleepers.

Characteristics	Good sleepers	Poor sleepers	*p* value
Numbers (%)	29 (36.30%)	51 (63.7%)	0.294^a^
Age (years old)	60.34 ± 13.849	58.53 ± 13.409	0.567
Gender			
Men	21 (72.4%)	31 (60.8%)	0.211^a^
Women	8 (27.6%)	20 (39.2%)	
Weight (kg)	71.03 ± 10.456	72.63 ± 9.50	0.489
Hemoglobin (g/dl)	11.86 ± 1.326	11.58 ± 1.462	0.406
Vitamin D (ng/ml)	26.45 ± 12.371	21.65 ± 10.425	0.101
Calcium (mg/dl)	8.72 ± 1.005	8.82 ± 0.445	0.530
Phosphate (mg/dl)	4.52 ± 0.826	4.89 ± 1.133	0.135
Calcium × phosphate	39.6059 ± 8.462	43.2298 ± 10.622	0.121
PTH (pg/ml)	337 (54–932)	367 (72–1298)	0.430^b^
Duration of HD (months)	5.50 (0–15)	4 (1–20)	0.560^b^
*Mode of HD*			
AV fistula	18 (62.1%)	35 (68.6%)	0.332^a^
CVC	10 (34.5%)	16 (31.4%)	
AV graft	1 (3.4%)	0 (0.0%)	
*Causes of ESRD*			
ADPKD	1 (3.4%)	1 (2.0%)	0.091^a^
CIN	3 (10.3%)	0 (0.0%)	
DM	0 (0.0%)	2 (6.9%)	
HTN	16 (55.2%)	30 (58.8%)	
HTN+DM	9 (31.0%)	18 (35.3%)	

Abbreviations: ADPKD: autosomal dominant polycystic kidney disease; AV fistula: arteriovenous fistula; AV graft: arteriovenous graft; CIN: chronic interstitial nephritis; CVC: central venous catheter; DM: diabetes mellitus; ESRD: end-stage renal disease; HD: hemodialysis; HTN: hypertension; PTH: parathyroid hormone. The quantity data are expressed as mean ± SD. ^a^Numbers (%), chi-square test. ^b^Median (min–max), Mann–Whitney test.

**Table 2:** Correlations between the studied variables in bad sleepers with PSQI score > 5.

	Age	Weight	Hb	Vitamin D	Duration of HD	PSQI	Ca	P	PTH	Ca × P
Age, *r*	1	-0.391^∗∗^	-0.125	0.132	-0.182	-0.210	0.026	0.127	-0.140	0.127
*p* value		0.005	0.381	0.356	0.201	0.140	0.856	0.381	0.349	0.378
Weight, *r*	-0.391^∗∗^	1	0.181	-0.087	-0.015	0.084	-0.050	-0.033	0.155	-0.033
*p* value	0.005		0.203	0.543	0.915	0.558	0.731	0.819	0.299	0.819
Hemoglobin, *r*	-0.125	0.181	1	-0.086	0.085	0.199	0.212	-0.259	-0.123	-0.205
*p* value	0.381	0.203		0.549	0.553	0.161	0.140	0.070	0.411	0.154
Vitamin D, *r*	0.132	-0.087	-0.086	1	-0.085	-0.397^∗∗^	-0.061	-0.188	-0.187	-0.187
*p* value	0.356	0.543	0.549		0.553	0.004	0.673	0.191	0.209	0.194
Duration of HD	-0.182	-0.015	0.085	-0.085	1	0.141	-0.057	-0.122	0.155	-0.119
*p* value	0.201	0.915	0.553	0.553		0.324	0.696	0.397	0.298	0.0410
PSQI, *r*	-0.210	0.084	0.199	-0.397^∗∗^	0.141	1	0.024	-0.153	-0.127	-0.142
*p* value	0.140	0.558	0.161	0.004	0.324		0.867	0.288	0.394	0.327
Calcium, *r*	0.026	-0.050	0.212	-0.061	-0.057	0.024	1	0.199	-0.383^∗∗^	0.387^∗∗^
*p* value	0.856	0.731	0.140	0.673	0.696	0.867		0.167	0.008	0.006
Phosphate, *r*	0.127	-0.033	-0.259	-0.188	-0.122	-0.153	0.199	1	0.008	0.979^∗∗^
*p* value	0.381	0.819	0.070	0.191	0.397	0.288	0.167		0.959	0.000
PTH, *r*	-0.140	0.155	-0.123	-0.187	0.155	-0.127	-0.383^∗∗^	0.008	1	-0.064
*p* value	0.349	0.299	0.411	0.209	0.298	0.394	0.008	0.959		0.670
Ca × P, *r*	0.127	-0.033	-0.205	-0.187	-0.119	-0.142	0.387^∗∗^	0.979^∗∗^	-0.064	1
*p* value	0.378	0.819	0.154	0.194	0.410	0.327	0.006	<0.001	0.670	

Abbreviations: HD: hemodialysis; PSQI: Pittsburgh sleep quality index; PTH: parathyroid hormone.

**Table 3:** Association between vitamin D and sleep quality using linear regression modeling of HD patients.

Variables	*B*	Standard error	*t* value	*p* value^∗^
Vitamin D	-0.16	0.05	-3.41	**0.001**
Age	-0.041	0.048	-0.852	0.397
Weight	0.003	0.067	0.048	0.962
Hemoglobin	0.059	0.429	0.138	0.890
PTH	<0.001	0.00	-0.05	0.959
Calcium	0.417	0.866	0.482	0.632
Phosphate	-0.011	0.596	-0.018	0.986
Calcium × phosphate	<0.001	0.06	0.03	0.974

Abbreviations: *B*: unstandardized beta; PTH: parathyroid hormone. ^∗^*p* ≤ 0.05 was considered as significant result.

## Data Availability

The authors do agree to make all data and the statistical information available for review if required.
